# Supplementary material for: 3D printing of multi-scalable structures via high penetration near-infrared photopolymerization
Source: Nat Commun. 2020 Jul 10;11:3462. doi: 10.1038/s41467-020-17251-z (PMC7351743; doi:10.1038/s41467-020-17251-z)
Supplement: Supplementary file 1 — Supplementary Information [file 41467_2020_17251_MOESM1_ESM.pdf]

Supplementary Information

3D Printing of Multi-scalable Structures via High Penetration Near-Infrared Photopolymerization

Zhu et al.

Supplementary Methods

Materials: Photoinitiator titanocene (bis(cyclopentadienyl)bis[2,6-difluoro-3-(1-pyrryl)phenyl]titanium) was obtained from BASF (China) Co., Ltd. Difunctional bisphenol A epoxy acrylate oligomer (EA) RY1102A80 and trimethylolpropane acrylate (TMPTA) were supplied by Jiangsu Kailin Ruiyang Chemical (China) Co., Ltd. Silicon dioxide Ace Matt TS100 was a product of Evonik (China) Co. Ltd. Yellow, red, blue and were provided by Jiangsu Kuangshun Photosensitivity New-material Stock Co. White pigment was a product of DuPont (China) Co., Ltd. UCNP ($NaYF_4$:Yb,Tm; mean diameter: 1385 nm; PDI: 0.396) was purchased from Shanghai Ziqi Chemical Technology Co., Ltd.

Ink preparation: The ink was prepared by mechanically mixing the mixture of titanocene (1.0 w.t.%), UCNP (1.0 w.t.%), TS100 (13.0 w.t.%), EA (42.5 w.t.%) and TMPTA (42.5 w.t.%). For colored inks, the composition contained pigment (yellow, red, blue or white; 0.5 w.t.%) titanocene (1.0 w.t.%), UCNP (1.0 w.t.%), TS100 (12.5 w.t.%), EA (42.5 w.t.%) and TMPTA (42.5 w.t.%). After weighing, the indigredients were evenly dispersed in resins utilizing a paste mixer (PM300V, TRILOS). All the inks were defoamed by centrifugation before using.

3D Printing methods: All of the 3D printing experiments were carried on a home-built DIW 3D printer, consisting of a computer-controlled 3-axis gantry platform, a high-pressure booster, micro nozzles with various diameters, and a newly proposed NIR (FC-W-980H-50W, Changchun New Industries Optoelectronics Technology Co.,Ltd.) curing module. Desired architectures were prepared through controlled extrusion of the ink onto glass or steel substrate, accompanying with controlled moves along the X and Y axes. Printing paths were compiled as parameterized G-code scripts and designed to maximize continuity within each printed layer. A coaxial ink extrusion module is equipped with a coaxial extruder, shell material reservoir, core material reservoir, and two separate ink supply modules as shown in **Supplementary Figure 2**.

For preparation of core-shell tubing, the coaxial extruder was made of stainless steel and its detailed dimensioned are 1.3 mm (outer layer diameter) and 0.5 mm (inner layer diameter), that was fixed through the whole study. Pneumatic dispensers were modified for handling with high ink viscosity with high pressure resolution 1kPa. All movements were programmed in LabVIEW.

Characterization: UV-Vis absorption spectra of titanocene and pigments were dispersed in acetonitrile employing a spectrophotometer model as EMCLAB EMC-61PC-UV, while the

emission spectrum of UCNP was measured in hexane by CARY Eclipse series fluorescence spectrophotometer. Tensile test was exhibited by Instron 5967X Universal Testing Systems. Conversion of functional groups were monitored by computing the decrease of characteristic absorption peak utilizing ATR-FTIR. Reat-time FTIR rheological analysis was performed with NIR light irradiation employing a setup fused with ATR-FTIR (Nicolet iS10 series, Thermal Fisher) and rheometer (HAAKE MARS60 equipped with Rheonaut annex, Thermal Fisher). In the case of real-time rheological analysis, a fixed sample volume of 300 μL of ink was injected into the gap (0.300 mm) and sheared with a strain of 5.00 %, a frequency of 1 Hz and a maximum torque of 8.0×10^4 μN·m at 25 °C with holding 300 s before measurement carried out. The conversion of functional groups ($K_{f,t}$) was calculated as followed equation:

$$K_{f,t} = \left(1 - \frac{P_{f,t}/P_{f,i}}{P_{r,t}/P_{r,i}}\right) \times 100\% \quad (1)$$

where $P_{f,t}$ and $P_{f,i}$ stands for integrated peak area of functional groups at different status or initial status, and $P_{r,t}$ and $P_{r,i}$ are integrated reference peak area of non-reactive groups at different status or initial status to exclude interference of physical factors.

Fine filament extrusion: Micro-scale filaments were extruded utilizing a 5 μm capillary nozzle. The ink was diluted in tetrahydrofuran (20 vol. % solvent) and filtered with a 0.46 μm needle filter. The filament was about 17 μm measured by an optical microscope.

Color robustness test: Honeycomb-shaped structures (15 × 30 × 5 mm) with different colors (blue/yellow/red/white) were printed for color robustness test. For thermal shock test, two sets of the sample underwent 10 circles from -20 °C to 150 °C. Afterwards, constant humidity test was performed for half of the structures where the relative humidity was remained 100% at 50 °C for 72 h. Another set of samples was immersed in acidic solutions (1 M HCl solution) separately for 120 h.

Supplementary Figures

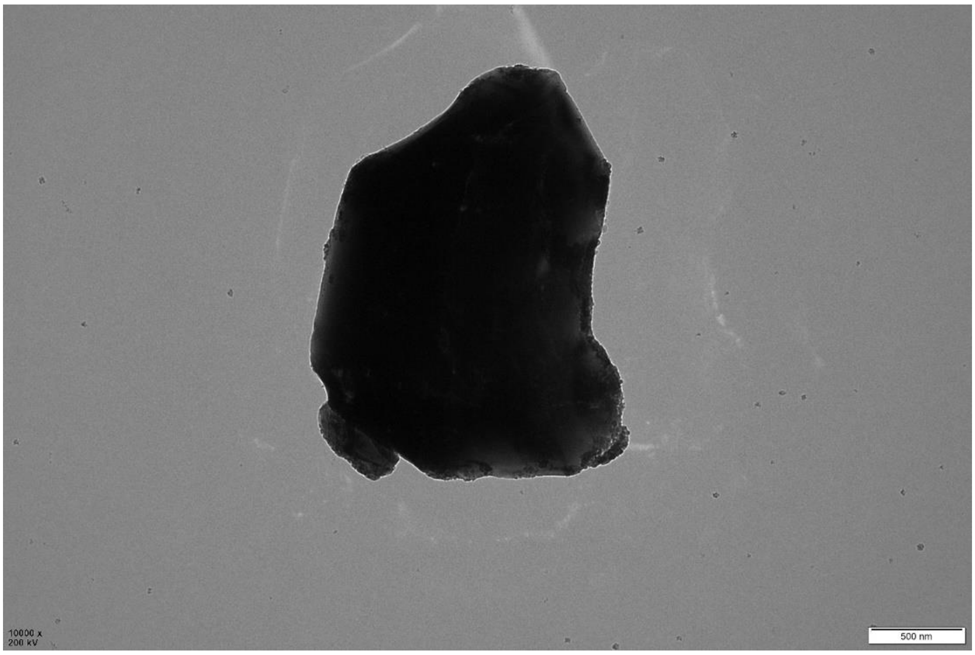

Supplementary Figure 1. TEM image of UCNP used in NIR-DIW inks

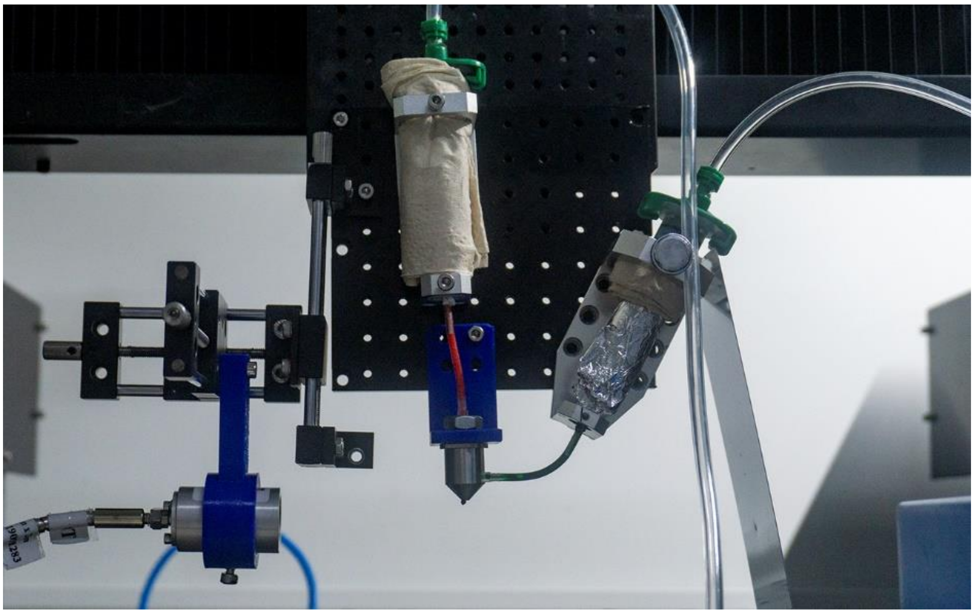

Supplementary Figure 2. Setup of coaxial extruder with NIR light source

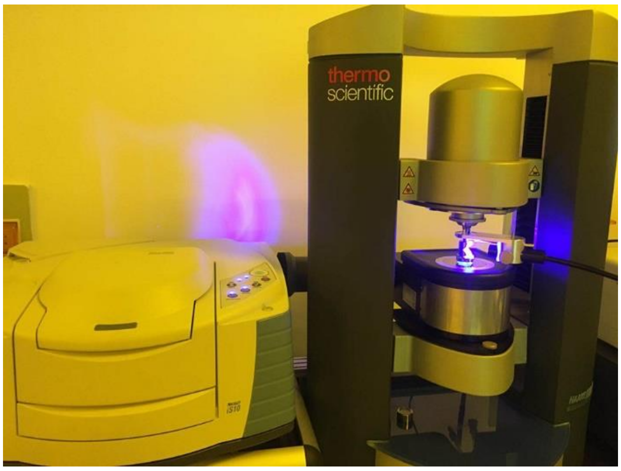

Supplementary Figure 3. Setup of ATR-FTIR and rheometer (blue light in the model was replaced by NIR light source in this case)

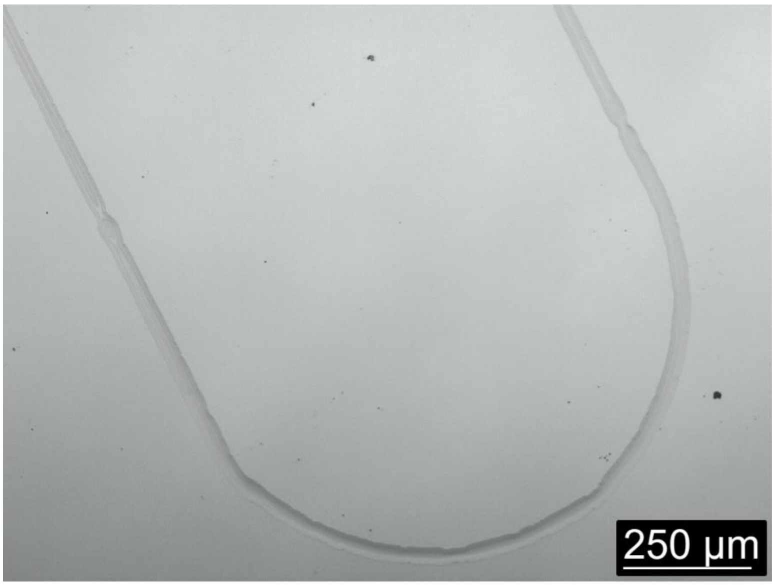

Supplementary Figure 4. Optical microscope image of filament extruded by capillary nozzle

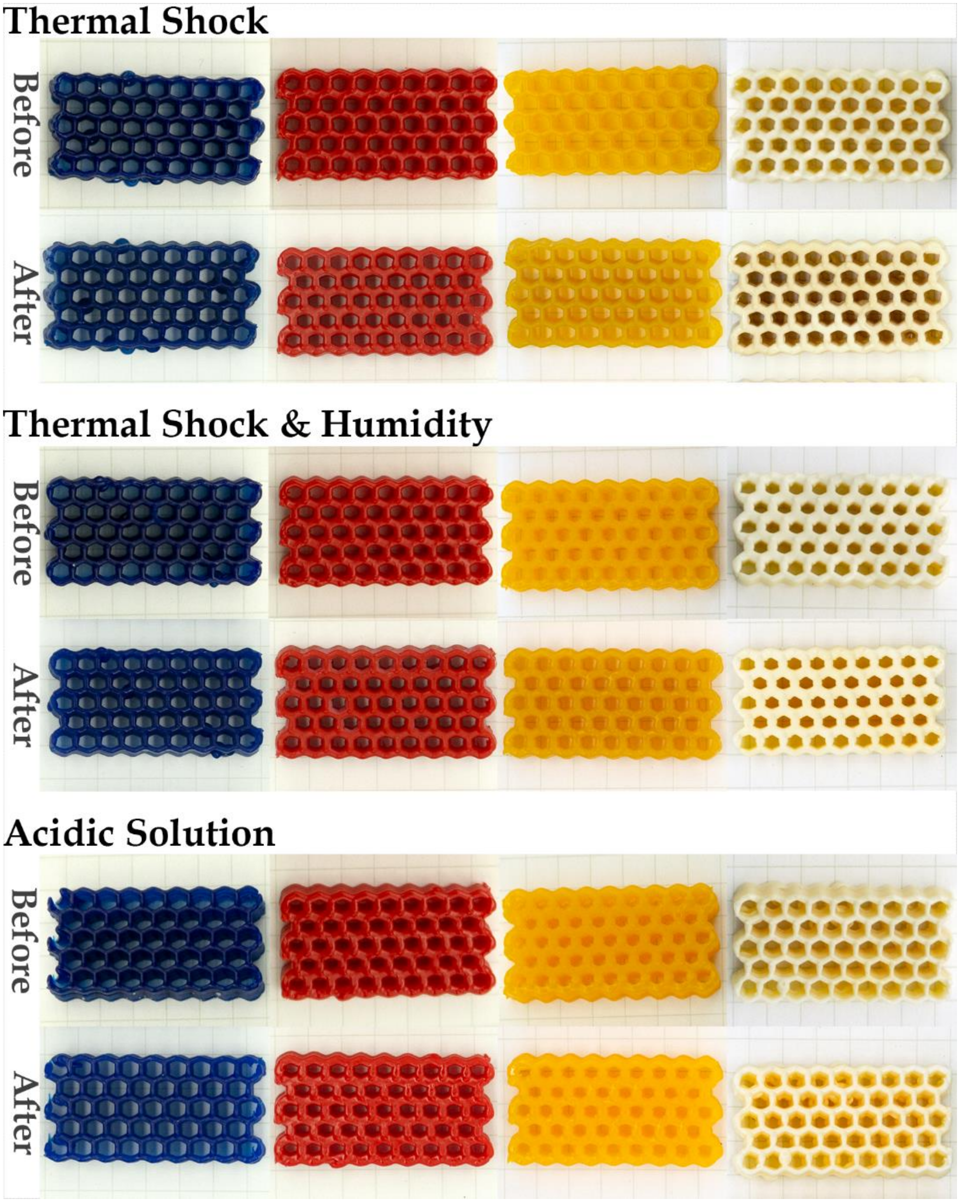

Supplementary Figure 5. Optical image of printed structure before and after color robustness test
